# The biological responses and mechanisms of endothelial cells to magnesium alloy

**DOI:** 10.1093/rb/rbab017

**Published:** 2021-05-28

**Authors:** Zhe Hou, Maolong Xiang, Nuoya Chen, Xiao Cai, Bo Zhang, Rifang Luo, Li Yang, Xiaoyi Ma, Lifeng Zhou, Fugui He, Hongchi Yu, Yunbing Wang

**Affiliations:** 1 National Engineering Research Center for Biomaterials, Sichuan University, Chengdu 610064, China; 2 College of Life Sciences, Sichuan University, Chengdu 610064, China; 3 The Fourth People’s Hospital of Chengdu, Chengdu 610036, China; 4 Beijing Key Laboratory of Cardiac Drug Device Technology and Evidence Based Medicine, Beijing 100021, China; 5 Institute of Biomedical Engineering, West China School of Basic Medical Sciences & Forensic Medicine, Sichuan University, Chengdu 610041, China

**Keywords:** magnesium alloy, endothelial cells, migration, adhesion

## Abstract

Due to its good biocompatibility and degradability, magnesium alloy (Mg alloy) has shown great promise in cardiovascular stent applications. Rapid stent re-endothelialization is derived from migrated and adhered endothelial cells (ECs), which is an effective way to reduce late thrombosis and inhibit hyperplasia. However, fundamental questions regarding Mg alloy affecting migration and adhesion of ECs are not fully understood. Here, we evaluated the effects of Mg alloy on the ECs proliferation, adhesion and migration. A global gene expression profiling of ECs co-culturing with Mg alloy was conducted, and the adhesion- and migration-related genes were examined. We found that Mg alloy had no adverse effects on ECs viability but significantly affected ECs migration and adhesion. Co-cultured with Mg alloy extract, ECs showed contractive adhesion morphology and decreased motility, which was supported by the down-regulation of adhesion-related genes (*Paxillin* and *Vinculin*) and migration-related genes (*RAC 1, Rho A* and *CDC 42*). Accordingly, the re-endothelialization of Mg alloy stent was inhibited *in vivo*. Our results may provide new inspiration for improving the broad application of Mg alloy stents.

## Introduction

Cardiovascular disease (CVD), which is characterized by high morbidity, high mortality and high disability rate, is the leading cause of death in the world [1]. Coronary heart disease counts for 43.2% of deaths attributable to CVD [2]. Coronary artery bypass graft (CABG) can completely vascularize the diseased part. However, after CABG surgery, the ratio of saphenous vein grafts occlusion arrives 10–25% from thrombosis within 1 year, and an additional 1–2% occlude from 1 to 5 years after CABG surgery [3]. Percutaneous transluminal coronary angioplasty with a coronary stent has been the routine treatment for coronary artery disease (CAD) [4]. However, in-stent restenosis (ISR) appears as the main problem after the bare-metal stent (BMS) implantation [5, 6]. Increasing evidence shows that intimal neogenesis, which is marked by excessive vascular smooth muscle cells (VSMCs) proliferation, is the decisive contributor to ISR [7, 8]. Accordingly, the drug-eluting stent (DES) is applied for suppressing the excessive proliferation of VSMCs to alleviate ISR [9, 10]. Despite that, the loading drugs, such as rapamycin (sirolimus) and paclitaxel, repress the excessive proliferation of VSMCs, but they also induce dysfunction of endothelial cells (ECs), which contributes to the delayed re-endothelialization and late stent thrombosis (LST) [11–16]. Biodegradable materials may disappear completely, thus avoid some lifelong problems caused by permanent implants, including permanent physical irritation and local chronic inflammatory reactions [17]. Therefore, it attracts considerable attention in coronary stent application.

With the better combination of strength and ductility compared with biodegradable polymer materials, biodegradable metallic materials, including magnesium (Mg), ferrous (Fe) and zinc (Zn) based alloy, have been proposed as potential materials for coronary stent implantation [18]. With the higher intake dosage daily and similar biomechanical characters to natural tissues [19], Mg-based alloy (Mg alloy) has been successfully used in several clinical trials [20–22]. ECs play an essential role in maintaining the integrity of the vessel by preventing thrombosis and hyperplasia. Basically, arterial injury is an inevitable consequence of stent implantation. Therefore, early and persistent EC coverage of the stent by structurally and functionally normal ECs should be considered the priority after stent implantation [23]. Although the influence of Mg ions on the proliferation of ECs has been shown [24], the effect of Mg alloy on ECs migration and adhesion abilities, which controls the stent re-endothelialization, is still not well known.

Here, we investigated Mg alloy’s effect on ECs migration and adhesion abilities *in vitro* and *vivo*. It revealed that ECs cultured in Mg alloy extract showed contractive adhesion morphology and down-regulated motility, which was supported by the down-regulation of adhesion-related genes (*Paxillin* and *Vinculin*) and migration-related genes (*RAC 1, Rho A* and *CDC 42*). On the surface of implanted Mg alloy in rabbits, ECs showed less attachment. Collectively, our data indicated that the adhesion and migration of ECs were deregulated by Mg alloy, resulting in the retarded re-endothelialization.

## Materials and methods

### Cell culture

Human coronary artery endothelial cells (HCAECs) were purchased from ScienCell™ (San Diego, CA, USA) and cultured in endothelial cell medium (ScienCell™), at 37°C, saturated humidity and 5% CO_2_. The medium included 500 mL of basal medium, 25 mL of foetal bovine serum (ScienCell™), 5 mL of EC growth supplement (ECGS, ScienCell™) and 5 mL of penicillin/streptomycin solution (P/S, ScienCell™). The cells were used for subsequent experiments when they formed a single layer.

### Mg alloy preparation

Mg alloy discs (10 mm × 2 mm) and stents (Ø 2 × 18 mm) were obtained from Beijing Amsino Medical Co., Ltd. The Mg alloy component contained Gd 3.5–5.5 wt%, Y 1.5–4.5 wt%, Zn 0–2.0 wt%, Zr 0–2.0 wt%. The metal discs were polished with SiC paper and cleaned ultrasonically in an acetone bath for 10 min. Before cell seeding, the metal discs were sterilized.

### Mg alloy extract medium preparation

The metal discs were sterilized and put into a 50-mL centrifuge tube. The Mg alloy extract was prepared using a serum-free endothelial cell medium with the surface area of extract medium ratio of 1.25 cm^2^/mL [25, 26] and incubated in a 37°C incubator with supplementation of 5% CO_2_ for 7 days.

### Stent implantation

The New Zealand rabbits (3.0–3.5 kg) used in the present study were purchased from (DaShuo experimental animal Co. Ltd, Chengdu, China) and approved by the medical ethics committee of Sichuan University. Male rabbits were used in all *vivo* studies. Each stent was hand-crimped on a 3.0 mm angioplasty balloon and intervened into the artery from the proximal iliac artery and then deployed (8-atm balloon inflation for 45 s) in the artery, achieving an approximate balloon to artery ratio of 1.2:1. Ultrasonic imaging (VINNO 6LAB, Vinno, China) was used to validate the stent’s proper location. The rabbits received 40 mg of aspirin orally 24 h before surgery and daily after that. Euthanasia was performed at 7, 14 and 28 days (*n* = 3 in every group) after stent deployment.

### Subcutaneous implantation

For measuring the biodegradation of Mg alloy *in vivo*, subcutaneous implantation was conducted. The rats used in the present study were purchased from DaShuo experimental animal Co. Ltd (Chengdu, China) and approved by the Medical Ethics Committee of Sichuan University. All *in vivo* studies were conducted in male rats. The animals were kept at a constant temperature (21 ± 1°C) under 12/12-h light/dark cycle and had free access to water and standard chow. Briefly, freshly prepared Mg alloy discs were implanted into individual dorsal subcutaneous pockets. Animals were euthanized after implanting 7, 14 or 28 days.

### Cell proliferation assay

HCAECs were plated at a density of 2000 cells/μL in a 96-well plate (100 μL/well) and incubated in a 37°C incubator with supplementation of 5% CO_2_. After incubation for 24 h, Mg alloy extract medium (100 μL) was added into the well and incubated for another 24 h, the final dilution of extract medium was 50%. According to the manufacturer’s instruction, cell proliferation was assessed using the CCK-8 assay (Sigma-Aldrich, USA). CCK-8 solution was added equal to 1/10 the media volume, and the incubation time was 2 h. Then, 10 µL of 1% SDS was used to stop the colour development reaction. The absorbance at 450 nm was measured.

### Scanning electron microscope

The surface morphology of Mg alloy after degradation *in vivo* or *in vitr*o was viewed. The cell samples were fixed by 4% paraformaldehyde buffer after cultured 24 h on the Mg alloy discs and dehydrated by gradient concentrations (75%, 90% and 100%) of ethanol for 5 min each time and dried. Then, cell morphologies were viewed using a scanning electron microscope (SEM; JSM-5900LV, JEOL, Japan).

### Cell migration assay

The cells were seeded in a six-well plate and cultured in the medium without fetal bovine serum (FBS) for 12 h. A plastic cell scraper was used to mark the vertical damage area (about 200 μm width) on the cells in each well. The experimental group was cultured with Mg alloy extract diluted four times with medium. After 0, 24, 48 and 96 h, the healing of the scratches was observed under an inverted phase-contrast microscope (CK2, Olympus, Japan) and recorded. ImageJ software was used to analyse the images. Three parallel experiments were done, and data were presented as mean ± standard deviation (SD).

### Real-time quantitative polymerase chain reaction detecting system

Total RNA was extracted from the cells with or without treatment by using the TRIzol reagent (Invitrogen Company, USA) according to the manufacturer’s instruction. The quantity was checked by NANODROP (Thermo, USA). The first-strand cDNA was synthesized by reverse transcription using RNA as a template. Sequences of the primers are listed in Table 1 (Sangon Biotech Company, China). Then, SsoFast™ EvaGreen^®^ Supermix (Bio-rad, USA) was used in the reaction. The conditions of response were as follows: activation of the enzyme at 95°C for 30 s, denaturation at 95°C for 5 s, annealing at 60°C for 5 s and followed by polymerization at 72°C for 10 s for 35 cycles. The melt curve was from 65°C to 95°C. β-actin was used as an internal control gene to obtain the relative expression values according to the delta-Ct method.

**Table 1.** Oligonucleotide primers used for QRT-PCR (human) analysis

Target transcript	Primer sequence (5′-3′)
*Rho A* (forward)	5′-CTGTCCCAACGTGCCCATCATC-3′
*Rho A* (reverse)	5′-CACCGGCTCCTGCTTCATCTTG-3′
*RAC 1* (forward)	5′-TGGGAGACGGAGCTGTAGGT-3′
*RAC 1* (reverse)	5′-AGGACTGCTCGGATCGCTTC-3′
*CDC 42* (forward)	5′-GCCCGTGACCTGAAGGCTGTCA-3′
*CDC 42* (reverse)	5′-TGCTTTTAGTATGATGCCGACACCA-3′
*Paxillin* (forward)	5′-ACCAGCAGCCTCAGTCCTCATC-3′
*Paxillin* (reverse)	5′-GCACGGAGAGCCAACACTGTC-3′
*Vinculin* (forward)	5′-AAAAATGACAGGGCTGGTGGA-3′
*Vinculin* (reverse)	5′-GCAGCTCAGGTTCGTAATCGT-3′
*β-Actin* (forward)	5′-CCTGGCACCCAGCACAAT-3′
*β-Actin* (reverse)	5′-GGGCCGGACTCGTCATAC-3′

### Western blot

The cells in the high-speed growth phase were treated with a 4-fold dilution of the Mg alloy extract and medium for 24 h, washed with PBS three times and added RIPA cell lysate containing 1% protease inhibitor and 1% phosphatase inhibitor. A cell scraper was used to hang the adherent cells. The lysate was collected, vortexed and placed on ice for 30 min to be fully lysed. After 12 000 g refrigerated centrifugation for 10 min, the supernatant was transferred to a clean EP tube. The Micro BCA™ Protein Assay Kit (Thermo Scientific, USA) was used to measure the protein concentration of the samples, and then a 5× loading buffer was added. The samples were heated at 100°C for 15 min to denature the proteins. The proteins were separated by using 10% SDS-PAGE, and the same amount of total protein (20 μg) was added to the loading well. After the electrophoresis, the proteins were transferred to the PVDF membrane, which was blocked with 5% skim milk for 2 h, incubated with the primary antibody (Table 2) at 4°C overnight, then washed 3 times for 5 min with TBST buffer. The secondary antibody was used to incubate for 2 h at room temperature. After washing with TBST three times, the targeted proteins were visualized with enhanced chemiluminescence (ECL, Beyotime Biotechnology Co., China) in the Molecular Image ChemiDoc XRS^+^ system (Bio-Rad Laboratories Inc., USA).

**Table 2.** Primary antibody information

Antibody	Company	Catalog number	Description	Western blot	IF
Anti-β-actin (human)	Cell Signaling Technology	#3700	Mouse monoclonal	1:1000	
Anti-Ki67	Cell Signaling Technology	#9449	Mouse monoclonal		1:800
Anti-CDC 42	Abcam	ab187643	Rabbit monoclonal	1:10 000	1:200
Anti-Rho A	Abcam	ab187027	Rabbit monoclonal	1:5000	1:150
Anti-RAC 1	Abcam	ab33186	Mouse monoclonal	1:1000	1:50
Anti-Paxillin	Abcam	ab32084	Rabbit monoclonal	1:5000	1:200
Anti-Vinculin	Abcam	ab129002	Rabbit monoclonal	1:10 000	1:200

### Immunofluorescence staining

The HCAECs were made into a cell suspension and evenly seeded on 14 mm round cell slides. When the confluence reached 90%, it was treated with 4-fold diluted Mg alloy extract for 24 h. After washing three times with PBS for 3 min, the samples were fixed with 4% paraformaldehyde for 30 min at room temperature, and then the samples were blocked in 5% goat serum with 0.1% triton for 30 min at room temperature. Ki67 (1:200), Paxillin (1:200) and Vinculin (1:200) primary antibodies (Table 2) diluted with 5% goat serum were incubated overnight at 4°C. After washing three times with PBS, FITC-labelled goat anti-rabbit IgG secondary antibody (1:1000) and TRITC-labelled goat anti-mouse IgG secondary antibody (1:1000) were incubated at room temperature for 1 h. After washing three times with PBS, the 4′6′-diamidino-2-phenylindole (DAPI) was diluted with PBS at a ratio of 1:1000, added into the samples and incubated at room temperature for 10 min. After washing with PBS four times, the samples were observed under confocal laser scanning with a confocal microscope (CLSM, Zeiss, Germany).

### RNA-sequence

After culturing on the Mg alloy 24 h, the cells were collected. According to the manufacturer’s instruction, total RNA was extracted from the sample by using the TRIzol reagent (Invitrogen Company). Before preparing the sequencing library by total RNA, agarose gel electrophoresis was used to detect quality control, and NANODROP measured the concentration of total RNA. Agilent 2100 was used for library quality control that was quantified by qPCR. Then Illumina Hiseq 4000 was used for sequencing. After using FastQC software to detect sequence quality, the abundance of transcripts in each sample was estimated by StringTie. The FPKM value (≥0.5) of genes and transcripts was assessed using the R package Ballgown. Then, differentially expressed genes and transcripts were filtered by R package Ballgown, and correlation analysis was processed.

### Statistical analysis

All experimental data are expressed as mean ± SD. SSPS and Graphpad software were used for statistical analysis. Statistical significance was determined using one-way analysis of variance followed by Tukey’s test or two-tailed unpaired *t*-test. At least three independent experiments were performed for all biochemical experiments, and the representative images were shown. *P <* *0.05 denotes statistically significant difference compared to control; **P <* *0.01 denotes highly significant difference compare to control; ***P <* *0.001 denotes extremely significant difference compare to control; n.s. denotes no significant difference.

### Availability of data and material

Gene expression profile data have been deposited for public access in the NCBI Gene Expression Omnibus under Accession Number (GSE146167). All data needed to evaluate the conclusions in the paper are present in the paper. Additional data related to this article may be requested from the authors.

## Results and discussion

### The degradation of Mg alloy *in vivo* and *in vitro*

**Figure 1.**
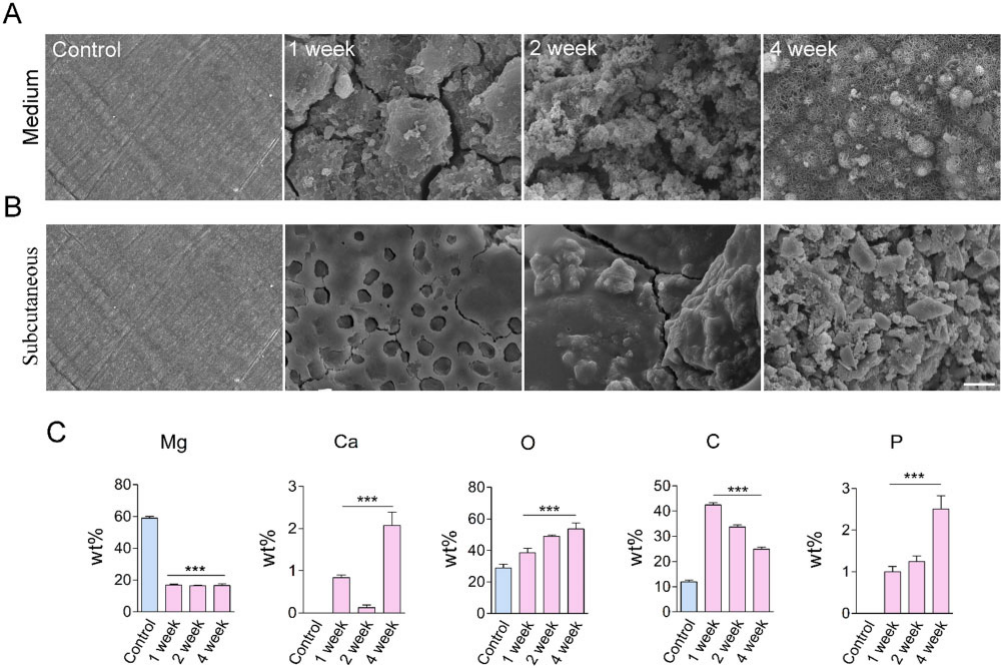
The degradation of Mg alloy *in vivo* and *in vitro*. (**A**, **B**) The surface morphologies of degraded discs in medium and subcutaneous tissue at different duration, scale bar = 50 μm. (**C**) EDS was used to measure the composition of Mg alloy surface elements after implanting in subcutaneous tissue at the indicated time. Data were presented as mean ± SD; statistics were performed using one-way analysis of variance followed by Tukey test, ***P <* *0.001.

The performance of an Mg alloy stent is primarily determined by its degradation *in vivo.* Therefore, we initially measured Mg alloy’s degradation in artificial plasma and subcutaneous tissue of male rats. In artificial plasma, Mg alloy’s corrosion was relatively faster than in subcutaneous tissue (Fig. 1A and B). It indicated that the Mg alloy used in this study had well corrosion resistance *in vivo*; it was lined with the successful application of Magmaris^®^ stent [22]. Energy dispersive spectrometer was used to measure the composition of Mg alloy surface elements after implantation in subcutaneous tissue at the indicated time. The concentration of the Mg element decreased on the surface (Fig. 1C); on the other hand, phosphorus (P) and calcium (Ca) had deposited on the surface (Fig. 1C). It suggested that the conversion of Mg phosphate to calcium phosphate occurred during the degradation of Mg alloy.

### Mg alloy had good biocompatibility for ECs

**Figure 2.**
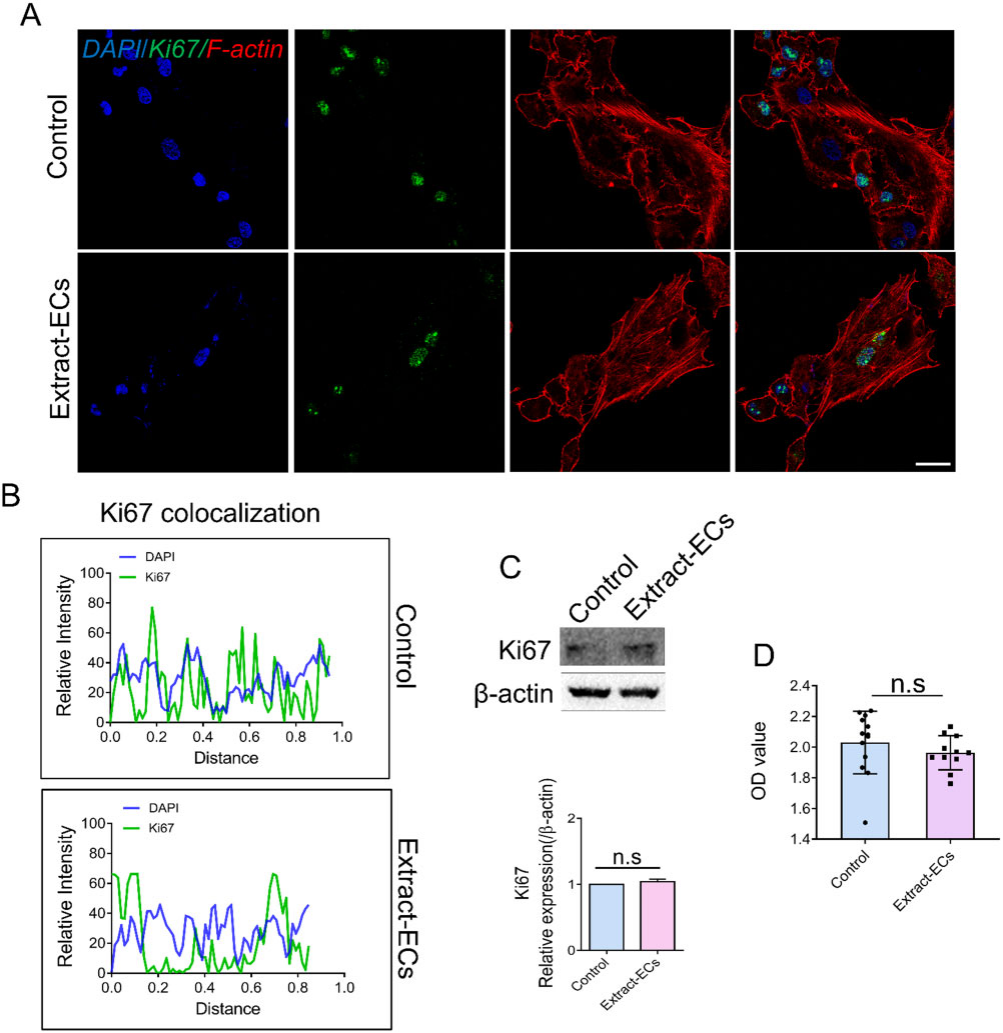
The effect of Mg alloy extract on the proliferation of endothelial cells. (**A**) The distribution and expression of Ki67 in HCAECs with or without Mg alloy extract medium treatment were observed by CLSM. (blue: DAPI; red: F-actin; green: Ki67; scale bar = 10 μm). (**B**) Co-localization of Ki67 and nuclear was measured by ImageJ software. (**C**) The protein expression of Ki67 was detected by western blot analysis. β-Actin was used as the internal control (*n* = 3). (**D**) The viability of HCACEs was examined by using CCK-8. Data are presented as mean ± SD; statistics were performed by two-tailed unpaired *t*-test, n.s. denotes not significant.

To further validate the biocompatibility of Mg alloy, we conducted immunostaining and western blot to examine the expression and location of Ki67, which was relevant to cell proliferation [27]. The location of Ki67 in HCAECs after exposing to Mg alloy extract medium (thereafter was referred to as Extract-ECs, HCAECs cultured in the standard medium was referred to as Control) was detected, and no translocation was observed (Fig. 2A and B). Western blot band showed that the expression of Ki67 had no significant difference (Fig. 2C). It revealed that Ki67 had a stable expression and location in HCAECs with or without extract medium treatment. Furthermore, the viability of Extract-ECs was measured by CCK-8 analysis. The viability of Extract-ECs was similar to the Control group (Fig. 2D). Collectively, the results showed that Mg alloy had good biocompatibility to HCAECs.

### Global gene expression profile of HCAECs subjecting to Mg alloy

**Figure 3.**
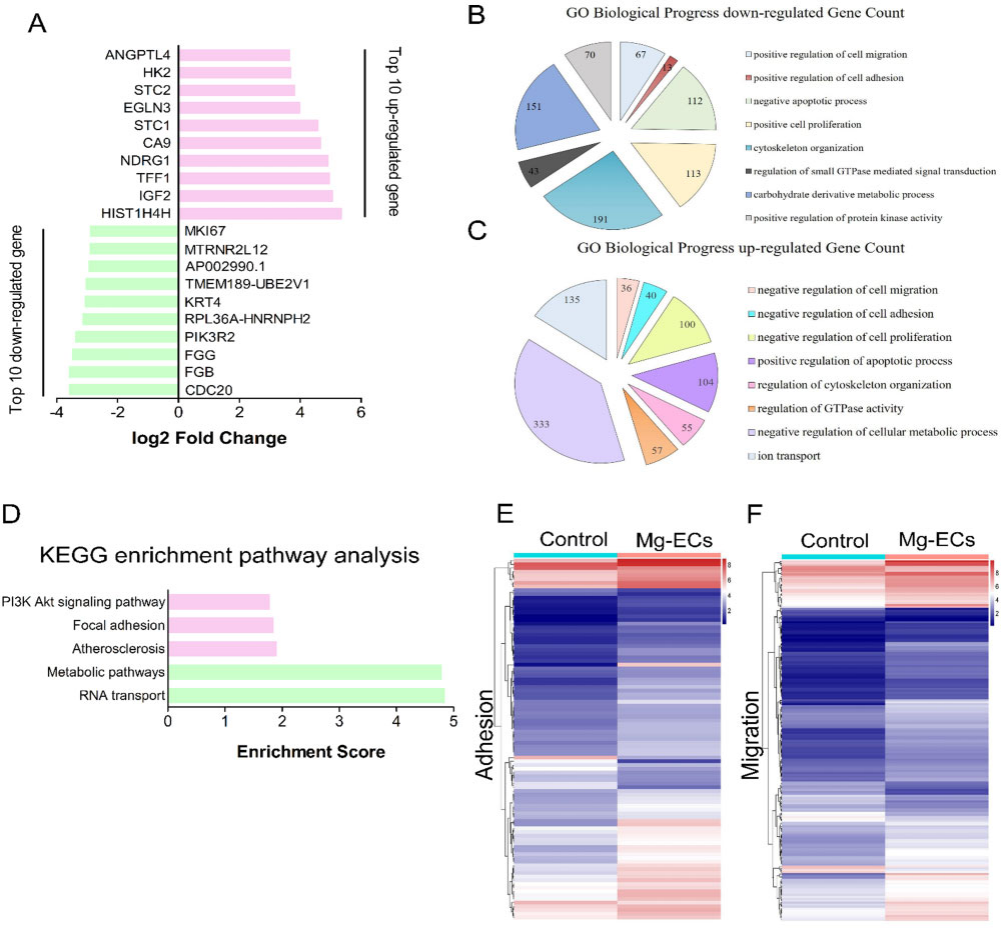
Mg alloy regulated gene expression associated with adhesion and migration. (**A**) Top 20 significantly changed genes detected by RNA-seq in HCAECs seeded on Mg alloy discs compared with HCAECs (up-regulated coloured red, down-regulated coloured green). (**B**, **C**) BP analysis of differently expressed genes in HCAECs seeded on Mg alloy discs compared with HCAECs. (**D**) KEGG enrichment pathway analysis for changed pathway associated with adhesion and migration in HCAECs seeded on Mg alloy discs compared with HCAECs. (**E**, **F**) Differently expressed genes involved in adhesion and migration detected by RNA-seq were shown in the hierarchical diagram in HCAECs seeded on Mg alloy discs compared with HCAECs.

We next sought to investigate how the Mg alloy dysregulated the migration and adhesion of HCAECs. Therefore, we conducted global gene expression profiling of HCAECs cultured on Mg alloy discs after 24 h (referred to as Mg-ECs, cells cultured on the dish are referred to as Control). The top 20 significantly changed genes are shown in Fig. 3A. Biological process (BP) enrichment analysis revealed the down-regulated adhesion and migration ability of HCAECs affected by Mg alloy (Fig. 3B and C). Kyoto Encyclopedia of Genes and Genomes enrichment analysis showed signal pathways related to cell adhesion and migration changed significantly (Fig. 3D). Heat map showed the differently expressed genes involved in adhesion and migration (Fig. 3E and F); the detailed gene list is shown in Tables 3 and 4.

**Table 3.** The migration genes in heat map

Gene name	Fold change (up-regulated)	Gene name	Fold change (up-regulated)
*STC1*	24.27777	*FLCN*	2.685
*IGFBP3*	13.64642	*CIB1*	2.637667
*CEMIP*	10.31747	*HRH1*	2.636922
*AGTR1*	10.01508	*TP53INP1*	2.633842
*CITED2*	8.278024	*LGALS3*	2.626668
*VEGFA*	7.57256	*HBEGF*	2.609182
*SERPINE1*	7.133048	*MDM2*	2.599837
*LOXL2*	6.570949	*MATN2*	2.587203
*NOV*	5.729413	*PRSS3*	2.547352
*BTG1*	5.496675	*ARHGEF2*	2.545455
*MMP1*	5.404093	*CD58*	2.531493
*ATP1B1*	5.13255	*CXCL8*	2.487225
*JUN*	4.843613	*KRT16*	2.465552
*F3*	4.646604	*SLC16A3*	2.458289
*TNS1*	4.501898	*SOX9*	2.456554
*L1CAM*	4.325589	*SNAI2*	2.442667
*DNER*	4.096347	*DDR1*	2.440238
*TNFRSF10D*	3.979881	*CCL20*	2.375038
*SPOCK1*	3.970137	*C16orf45*	2.365153
*ITGA2*	3.893582	*SPRY2*	2.35184
*TNFAIP6*	3.857849	*SCARB1*	2.341672
*LAMC2*	3.687884	*SFRP1*	2.339137
*CXCR4*	3.580353	*BMP2*	2.333049
*IRS2*	3.526425	*SP100*	2.330621
*TNFSF12*	3.316922	*ACVR1*	2.323221
*LRP1*	3.315323	*TNFRSF10B*	2.288293
*PRKCA*	3.285778	*DDIT4*	2.256568
*SDC4*	3.201197	*SEMA4B*	2.25138
*SBK2*	3.19182	*HDAC5*	2.248255
*KLF4*	3.17761	*ITGB3*	2.243382
*MYADM*	3.162549	*AXL*	2.195178
*SH3KBP1*	3.146679	*PDGFA*	2.182462
*SPNS2*	3.128461	*RHOB*	2.175154
*ITGA5*	3.119477	*VEGFC*	2.153568
*TGFBR1*	3.118993	*SDC2*	2.147865
*ITGB4*	3.093541	*TGFB1*	2.146995
*NR4A2*	3.081555	*CEND1*	2.146292
*C5AR1*	3.048049	*TRIB1*	2.138017
*PDGFB*	3.040635	*APP*	2.120378
*PTK6*	2.872739	*BST2*	2.099677
*EGFR*	2.821483	*PTGS2*	2.095865
*COL5A1*	2.787228	*BSG*	2.092701
*ITGA3*	2.785423	*VEGFB*	2.079217
*GPI*	2.769742	*BMP4*	2.076694
*DUSP10*	2.72474	*TREM1*	2.068193
Gene name	Fold change (up-regulated)	Gene name	Fold change (up-regulated)
*DPP4*	2.057136	*NDEL1*	1.592378
*S100P*	2.051367	*RHOG*	1.58564
*MCTP1*	2.032091	*TNFRSF10A*	1.584607
*SMAD7*	2.031479	*PDPK1*	1.57535
*FGF18*	1.979253	*NRP1*	1.568169
*GPX1*	1.973924	*INSR*	1.563841
*SPRED1*	1.960228	*TNFRSF11A*	1.563333
*FOXC2*	1.954102	*AUTS2*	1.554992
*MET*	1.92322	*RPS19*	1.55284
*F2RL1*	1.923216	*SBDS*	1.552702
*NDNF*	1.887539	*ZNF703*	1.551496
*IL1A*	1.884756	*IGFBP5*	1.547377
*CD63*	1.859589	*ARC*	1.546084
*DMTN*	1.83036	*RHBDF1*	1.537457
*BCAR1*	1.81994	*PLXNB3*	1.534637
*MBOAT7*	1.816693	*BAMBI*	1.514972
*CCL5*	1.803252	*DPYSL3*	1.512534
*TIMP1*	1.787133	*PSEN1*	1.510164
*PLXNA2*	1.776413	Gene name	Fold change (down-regulated)
*SPHK1*	1.772645	*HIF1A*	0.240812
*FAM89B*	1.76988	*SEMA6B*	0.255757
*TNFRSF12A*	1.763901	*PKN3*	0.258733
*RTN4*	1.761169	*EMP2*	0.318825
*MAPK14*	1.755461	*KIF20B*	0.333759
*STK24*	1.742037	*SHTN1*	0.348257
*SDCBP*	1.738339	*ANLN*	0.363631
*CYP1B1*	1.737953	*PLXND1*	0.367796
*MIF*	1.734608	*IL1R1*	0.401752
*APC*	1.734293	*NUP93*	0.411265
*MAP3K3*	1.699488	*AMOTL2*	0.416412
*GNRH1*	1.695838	*ID1*	0.417674
*RAP2B*	1.68903	*HMGB1*	0.422201
*SLC9A1*	1.688039	*C1QBP*	0.450745
*PODXL*	1.67678	*STAT1*	0.451953
*S100A2*	1.672244	*SMO*	0.452743
*ZMYND8*	1.66521	*CENPV*	0.452901
*SELPLG*	1.661614	*NUP188*	0.467387
*ANO6*	1.653642	*SEMA3A*	0.502904
*NOG*	1.647961	*SEMA4G*	0.507607
*GREM1*	1.645635	*CXCL5*	0.536764
*DDRGK1*	1.64124	*ZNF609*	0.544623
*ITGAX*	1.634522	*JAM3*	0.546291
*ARHGAP4*	1.633223	*EDN1*	0.553224
*BMPR2*	1.6207	*NRTN*	0.57845
*NDRG4*	1.61812	*SEMA3C*	0.579641
*RAB13*	1.61544	*PAXIP1*	0.616381
Gene name	Fold change (down-regulated)	Gene name	Fold change (down-regulated)
*ADGRA2*	0.628179	*GIPC1*	0.642236
*PF4*	0.635523	*PKN2*	0.644713
*TMEM201*	0.638581	*LBP*	0.648244
*FERMT1*	0.640175	*CXCL2*	0.652023

**Table 4.** The adhesion genes in heat map

Gene name	Fold change (up-regulated)	Gene name	Fold change (up-regulated)
*IGF2*	33.53979	*SMAD7*	2.031479
*CITED2*	8.278024	*EFNA5*	2.01906
*VEGFA*	7.57256	*HLA-E*	2.006855
*SERPINE1*	7.133048	*ZBTB7B*	2.001766
*SERPINE2*	4.271194	*FOXC2*	1.954102
*ARG2*	4.040669	*SOCS5*	1.944846
*SPOCK1*	3.970137	*STX3*	1.893833
*ITGA2*	3.893582	*NDNF*	1.887539
*PLXNA3*	3.827364	*S100A10*	1.859606
*MUC1*	3.437202	*ADORA2A*	1.842497
*CD55*	3.315238	*DMTN*	1.83036
*PRKCA*	3.285778	*CCL5*	1.803252
*SDC4*	3.201197	*PLXNA2*	1.776413
*KLF4*	3.17761	*EBI3*	1.742559
*MYADM*	3.162549	*CYP1B1*	1.737953
*ITGA5*	3.119477	*SOCS1*	1.726588
*ZBTB1*	3.051579	*CD164*	1.720611
*ITGA3*	2.785423	*IGFBP2*	1.710745
*RUNX1*	2.773166	*GNRH1*	1.695838
*PPP1CB*	2.733251	*SLC9A1*	1.688039
*DUSP10*	2.72474	*MAPK7*	1.680037
*FLCN*	2.685	*PODXL*	1.67678
*CIB1*	2.637667	*GREM1*	1.645635
*LGALS3*	2.626668	*FXYD5*	1.639516
*CXCL8*	2.487225	*TNFSF9*	1.616681
*SOX9*	2.456554	*CD9*	1.600961
*SNAI2*	2.442667	*PDPK1*	1.57535
*DDR1*	2.440238	*PRKAR1A*	1.571276
*TFRC*	2.403466	*DUSP3*	1.569103
*CD276*	2.355959	*NRP1*	1.568169
*PAG1*	2.354704	*LPXN*	1.566536
*LMO7*	2.342407	*DHPS*	1.559871
*SFRP1*	2.339137	*ZNF703*	1.551496
*BMP2*	2.333049	*PLXNB3*	1.534637
*LGALS1*	2.259964	*AGER*	1.530005
*RND1*	2.164827	*TSC1*	1.526465
*VEGFC*	2.153568	*RPS3*	1.517731
*TGFB1*	2.146995	*TESC*	1.507311
*ACER2*	2.128129	*BCL6*	1.506574
*CYTH1*	2.093908	Gene name	Fold change (down-regulated)
*FSTL3*	2.080918	*MAD2L2*	0.64414
*BMP4*	2.076694	*FERMT1*	0.640175
*BCL10*	2.072502	*ADAM9*	0.595791
*RARA*	2.065234	*PTPN6*	0.568468
*DPP4*	2.057136	*GSTP1*	0.556946
Gene name	Fold change (down-regulated)	Gene name	Fold change (down-regulated)
*GCNT2*	0.543328	*FGA*	0.32608
*SKP2*	0.507553	*EPCAM*	0.260552
*RAC3*	0.475456	*FGB*	0.131485
*TGFB2*	0.333832	*FGG*	0.128291

### Mg alloy inhibited the adhesion ability of HCAECs

**Figure 4.**
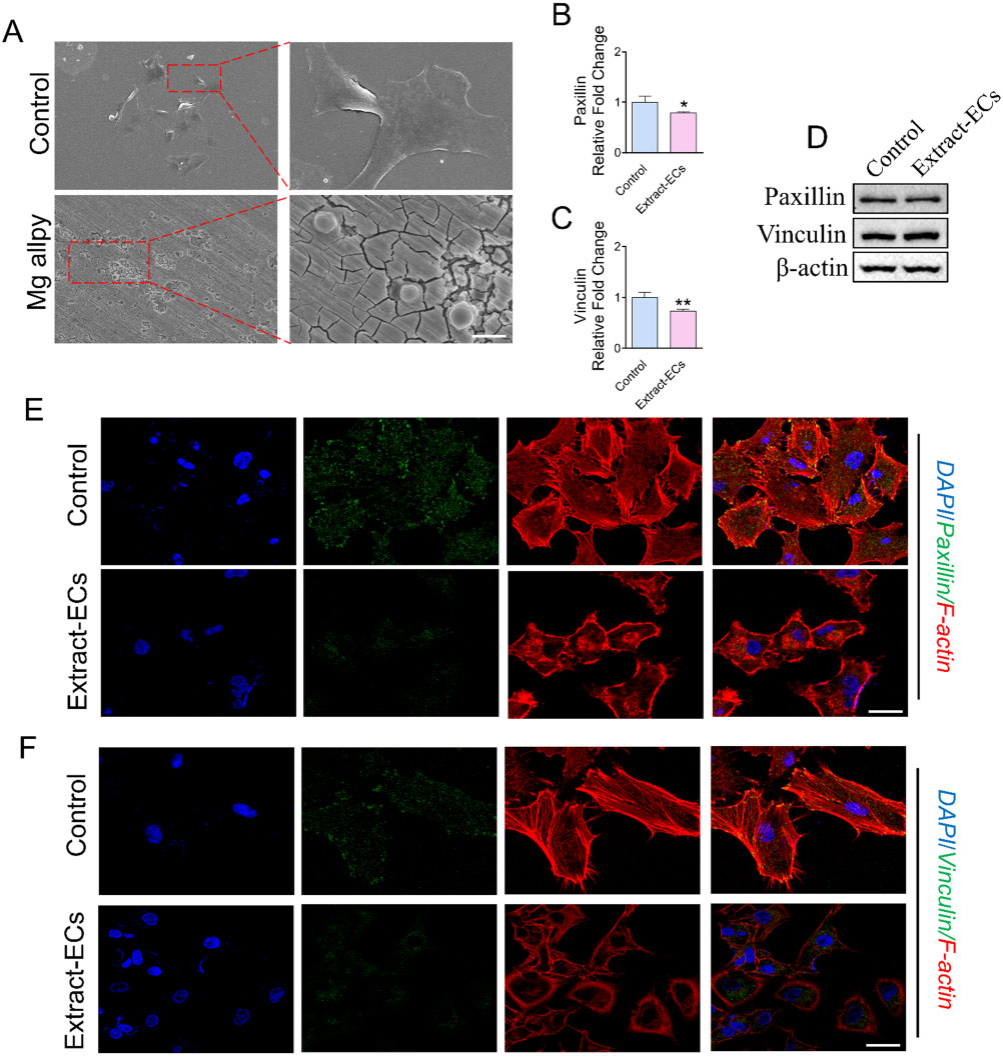
The effect of Mg alloy on HCAECs adhesion. (**A**) SEM images showed the adhesion morphology of HCAECs on the Mg alloy disc. (**B**, **C**) The results of qRT-PCR for *Paxillin* and *Vinculin* (*n* = 3). *P <* *0.05; **P <* *0.01. β-actin was used as the internal control (*n* = 3). data are presented as mean ± SD; statistics were performed by two-tailed unpaired *t*-test. (**D**) Western blot analysis was used to measure the expression of focal adhesion protein (Paxillin and Vinculin); β-actin was used as the internal control. (**E**, **F**) The distribution and expression of Paxillin and Vinculin in HCAECs with or without Mg alloy extract-treatment were detected by immunostaining (blue: DAPI; red: F-actin; green: Paxillin/Vinculin; scale bar = 10 μm).

To validate the results from RNA-seq, we examined adhesion morphology and expression of adhesion-related genes in Extract-ECs. HCAECs exhibited a spreading morphology cultured in dishes, while it had contractive morphology when seeded on Mg alloy discs (Fig. 4A). Focal adhesions (FAs) are required for cells spreading and adhesion. Accordingly, we measured the dynamics of several core FAs proteins. Adhesion proteins can be separated into distinct ‘modules’ based on their different functions [28]. The ‘signalling module’ protein Paxillin was down-regulated at the gene level while had a faint change at the protein level (Fig. 4B and D). As the ‘structural module’, Vinculin, which is essential for cell adhesion and spreading, decreased at the gene level and elevated little at protein level (Fig. 4C). It was in agreement with the contractive morphology of HCAECs seeded on the Mg alloy discs. The immunostaining further confirmed the down-regulated expressions of Paxillin and Vinculin (Fig. 4E and F).

### Mg alloy reduced the migration ability of ECs

**Figure 5.**
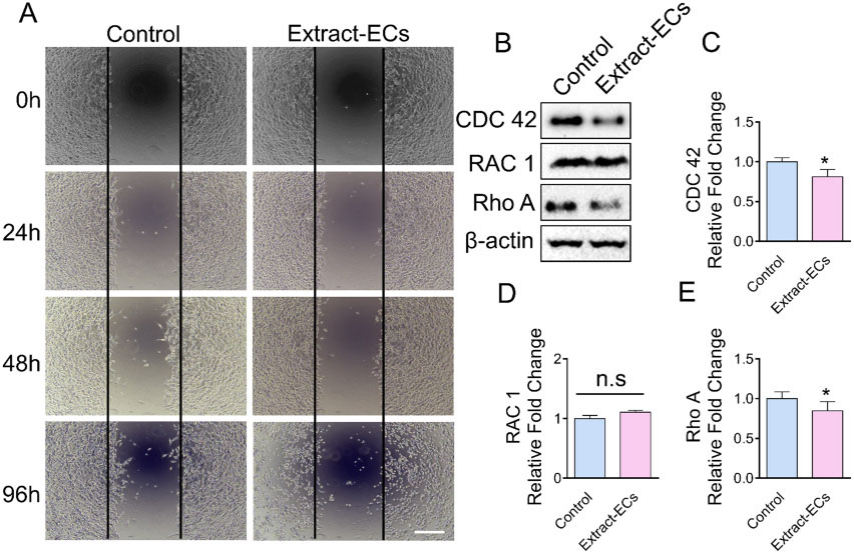
The Effect of Mg alloy extract on HCAECs migration. (**A**) Wound healing assay shows the difference of cell motility between HCAECs and Extract-ECs at the indicated time, scale bar = 100 μm. (**B**) The expressions of migration-related proteins (CDC 42, RAC 1 and Rho A) were detected by Western blot. (**C–E**) The mRNA expressions of *CDC 42*, *RAC 1* and *Rho A* were examined by qRT-PCR. Data are presented as mean ± SD; statistics were performed by two-tailed unpaired *t*-test, β-actin was used as the internal control in western blot and qRT-PCR analysis, respectively (*n* = 3), *P <* *0.05.

Aiming to investigate Mg alloy’s effect on HCAECs motility, we performed a wound-healing assay and found that Mg alloy repressed HCAECs motility. The Mg alloy extract medium retarded the migration of HCAECs compared with the normal medium (Fig. 5A). RAC 1, Rho A, CDC 42 are essential members in Rho GTPases and control cell motility [29]. Western blot was conducted to measure the expression of RAC 1, Rho A and CDC 42 in Extract-ECs. The results showed that Mg alloy reduced the expressions of CDC 42 and Rho A, while RAC 1 had stable expression (Fig. 5B). It was supported by the real-time quantitative polymerase chain reaction (qRT-PCR) expression results (Fig. 5C–E). The above data indicated that Mg alloy down-regulated Rho GTPases’ expression, which resulted in the decreased migration ability.

### Mg alloy stent retarded the complete re-endothelialization *in vivo*

**Figure 6.**
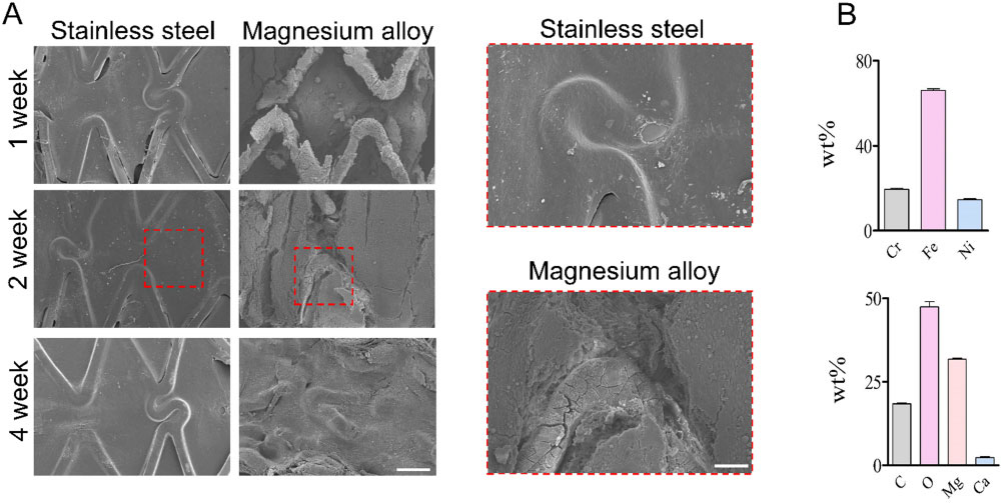
The Re-endothelialization condition of the Mg alloy stent after implantation. (**A**) SEM images showed the re-endothelialization of the stent at different time durations, scale bar = 500 μm (left panel), 200 μm (right panel). (**B**) EDS images showed the surface chemical element composition change of stents at 2 weeks.

Rapid EC coverage of the stent is required for avoiding thrombosis and late restenosis. Here, we conducted stent implantation in the rabbit artery to access the re-endothelialization of Mg alloy stent. A completed ECs layer was mainly finished in 2 weeks on stainless steel stent, while re-endothelialization was suppressed by the Mg alloy stent (Fig. 6A). Mg alloy’s degradation caused the composition change of the stent surface, which might arrive at an unfriendly ion level for the ECs (Fig. 6B).

## Conclusions

Percutaneous coronary artery intervention associated with stents is most widely accepted as an effective and safe treatment for single vessel and multi-vessel coronary atherosclerotic disease. Although BMS impedes restenosis dramatically compared to balloon angioplasty in the last decades, restenosis incidence still arrives 20–30% [30]. Therefore, DES had been used, and the rate of restenosis (5–10%) could be lower than BMSs [30]. The metallic materials of BMS and DES were kept in the vessel forever, although the drug was eluted and the polymer coat was degraded. Permanent vessel caging impairs arterial physiology, and the probability of very LST, though the occurrence probability is very low [31]. Because of its degradation properties in the physiological environment, the degradable stent was recently attracted much attention.

As a new material with high biocompatibility and excellent mechanical properties, the degradable Mg alloy material has been focussed on its potential application in the generation of biodegradable stents [17]. The rapid corrosion of Mg alloy material is one of the main constraints to its clinical application, although the Magmaris^®^ stent has been successfully used [22]. Therefore, extensive studies have focussed on inhibiting stent corrosion and improving the mechanical properties of Mg-based alloy stents [32]. The main degradation product, Mg^2+^, which is one of the most abundant intracellular cations, takes part in various vital cellular reactions to influence the viability and proliferation of HCAECs [33]. The Mg salt solution, such as MgCl_2_, was used to measure the effect of Mg alloy degradation elements on vascular cells [24]. However, a variable concentration of Mg salt solution was used in the past research [34]. It is not easy to specify the effect of Mg salt solution on the BP by choosing a specific concentration. On the other hand, ECs cultured in the Mg salt solution does not simulate the *in vivo* situation as the ECs directly contact the Mg alloy materials *in vivo*. Therefore, we cultured ECs exposed to Mg alloy discs or extract medium 24 h to avoid the damage of degradation products in the constant pool to ECs. The genes got enough time to express differently and then keep relatively stable. Furthermore, the pH shift in Mg corrosion is another factor that may regulate gene expression of ECs [35]. Meanwhile, pH change happened during the cell culturing process [36]. The underlying biomolecular mechanism by which the pH shift in Mg corrosion regulates ECs gene expression is not well known.

In this study, we evaluated the effects of Mg alloy on the cellular responses of HCAECs. The rate of re-endothelialization depends on the ability of the cells to adhere, migrate, proliferate and so on [23]. We found that after 24-h incubation with Mg-Extract, the viability and proliferation rate of HCAECs had no significant adverse effects (Fig. 2). The expression proﬁles of genes related to cell adhesion and migration were altered when HCAECs were seeded on Mg alloy discs (Fig. 3B and C). We further investigated the change of cell morphology after seeding directly on the Mg alloy discs. HCAECs tend to be round when seeded on Mg alloy discs (Fig. 4A). The morphology changes might also indicate that degradation products of Mg alloy inhibited the spreading process. The expression of FAs components, including Paxillin and Vinculin, was detected, and our results indicated that their expressions were decreased (Fig. 4B–D). In addition, the extract-ECs showed decreased motility (Fig. 5A), which was associated with down-regulated migration-related genes (Fig. 5B–E). The results of *in vivo* experiments also indicated that re-endothelialization of the Mg alloy stent surface was not perfected. Taken together, we hypothesized that Mg alloy exacerbated the process of re-endothelialization by affecting the migration and adhesion of ECs, but the underlying mechanism remained to be studied.

Mg alloy is bioresorbable scaffold material, which is being investigated for medical applications because of its enhanced properties of biodegradability and biocompatibility, such as bone replacement [18, 37]. However, there are few studies about the use of Mg alloy in CVDs, such as atherosclerosis (AS), which has been claimed to be the most common cause of death worldwide [38]. One of the limitations of using Mg -based alloy is the uncontrollability of degradation *in vivo* because of the electrochemically active property of Mg alloy [39]. At present, researchers have done many kinds of studies on corrosion resistance. Biodegradable polymer coatings are used to provide temporary corrosion resistance to Mg alloy for both orthopaedic and cardiovascular applications [40]. Here, we investigate the potential application of Mg alloy from a biomolecular view. The gene expression profiles showed that Mg alloy pronouncedly altered the expression of genes related to cell adhesion and migration. However, Mg alloy did not inhibit the proliferation of ECs. It indicated that the delayed re-endothelialization caused by Mg alloy was likely dependent on the retarded migration and adhesion ability of ECs. Our results may provide new inspiration for improving the broad application of Mg alloy stent in coronary AS.
